# Substance Use Right Before or During Work Among the Young US Workers: Evidence From the National Longitudinal Survey of Youth 1997 Cohort

**DOI:** 10.1002/ajim.23737

**Published:** 2025-05-15

**Authors:** Sehun Oh, Daejun Park, Sarah Al‐Hashemi

**Affiliations:** ^1^ College of Social Work The Ohio State University Columbus Ohio USA; ^2^ College of Health Sciences and Professions Ohio University Athens Ohio USA; ^3^ College of Public Health The Ohio State University Columbus Ohio USA

**Keywords:** food preparation/serving, occupation, safety‐sensitive occupations, substance use in the workplace, United States

## Abstract

**Objective:**

Substance use right before or during work (hereinafter, “substance use in the workplace”) poses significant health risks to users, colleagues, and the public in the workplace. However, less clear are figures on recent prevalence, characteristics of those engaging in such behaviors, and variations across occupations. This study examines the prevalence of substance use in the workplace, individual and work‐related characteristics, and substance use risks across different occupations among a nationally representative sample of workers in their early 30 s—a period of heightened substance use.

**Methods:**

Data from the National Longitudinal Survey of Youth 1997 (NLSY97) were analyzed, focusing on 6155 respondents. Past‐month prevalence of substance use in the workplace (separately for any substance, alcohol, marijuana, and cocaine/hard drugs) was assessed overall and by occupation using the Census 2002 Standard Occupational Classification. Multivariable Poisson regression models tested associations between occupation and substance use, adjusting for sociodemographic and health‐related characteristics.

**Results:**

In the past month, 8.9% of workers reported any substance use in the workplace, including 5.9% for alcohol, 3.1% for marijuana, and 0.8% for cocaine/hard drugs. Prevalence was highest in food preparation/serving occupations, followed by safety‐sensitive occupations. Our models indicated higher risks for all types of substance use among food preparation/serving workers, higher alcohol use among white‐collar workers, and elevated alcohol and marijuana use in safety‐sensitive occupations.

**Conclusions:**

The substantial prevalence of workforce substance use among individuals in their early 30 s raises public health concerns, underscoring the need for workplace interventions addressing occupation‐specific patterns of alcohol and marijuana use.

## 1 Introduction

Substance use right before or during work—hereinafter “substance use in the workplace” following the definition in the seminal work by Frone [1, 2]—can pose significant threats to health and well‐being of users, coworkers, and the public in the workplace. By impairing cognitive and behavioral functionality, either directly or indirectly (e.g., through fatigue and hangovers), research indicates that substance use increases the risk of work‐related incidents such as falls, cuts, or fractures [3, 4, 5]. For instance, in 2022, 525 fatal workplace injuries were attributed to the nonmedical use of alcohol or drugs, a rate eight times higher than in 2012 [6]. In addition to the direct harm to workers, substance use in the workplace also incurs considerable economic and societal costs. Workplace incidents—like those involving individuals treated for nonfatal injuries in emergency departments—result in an average loss of 11 workdays per incident and a cost of $1590 per person, placing significant social and financial burdens on the businesses and healthcare system [7, 8].

Despite its risks, substance use in the workplace remains prevalent, particularly among young workforce, a group at peak risk for substance use [9]. Studies analyzing data from the 2000 s indicate that 6%–7% of workers consume alcohol during the workday, mostly during lunch breaks, with 9% report experiencing hangovers [2, 10, 11]. This results in an overall workplace alcohol use and impairment prevalence—encompassing both on‐the‐job drinking and post‐work hangovers—ranging from 13.8% and 15.2% [2, 10, 11]. Additionally, data from the early 2000 s National Survey of Workplace Health and Safety show that a total of 3.3% of workers report illicit drug use or intoxication during the workday, with 2.4% engaging in illicit drug use and 2.8% experiencing intoxication [10, 12]. Young workers, especially those in male‐dominated occupations, such as construction, trades, and transportation, are more likely to be exposed to workplace cultures that normalize or even encourage substance use [13]. This trend is particularly concerning for young workforces who may have developed substance use patterns in adolescence, placing them at greater risk for continued alcohol and drug use in the workforce [13].

The type of occupation—through various workplace contextual factors (e.g., substance use norms, availability, or other occupation‐specific risk factors, such as physical and mental stress, occupational injury/illness, and limited access to healthcare)—can contribute to variations in substance use in the workplace. While direct evidence on substance use in the workplace is limited, research indicates that individuals in certain occupations face elevated risks of alcohol or drug use. For alcohol use, workers in “high‐prestige” occupations such as management (due to increased exposure to alcohol‐involved work and leisure activities), and food preparation/serving occupations (due to greater access at the workplace and more permissive use norms) report greater risks [14, 15, 16, 17, 18]. Additionally, workers in physically demanding occupations—including construction, extraction, and other skilled trades—as well as those in food preparation/serving and arts/entertainment/recreation occupations are at increased risk for alcohol and illicit drug use [19, 20]. Data from the Behavioral Risk Factor Surveillance System indicate that, between 2013 and 2016, one in four construction and extraction workers reported binge drinking, with an average of 749 total annual binge drinks per person [18]. Furthermore, nearly one in five construction workers and 14% of installation/maintenance/repair workers in 2005‐2016 reported using illicit drugs in the past 30 days [19]. While overall and workplace substance use share similar predictors and contextual factors, research has also identified discrepancies in their predictors—such as certain personality traits, substance use outcome expectancies, and workplace characteristics [21]. These findings suggest that evidence based on overall substance use patterns by occupation may not be directly applicable to workplace substance use, highlighting the need for research specifically focused on substance use within the workplace context.

Prior research on substance use in the workforce has several limitations that hinder a comprehensive understanding of this public health issue. First, many studies rely on data from the 2000 s, which may not accurately reflect recent trends in substance use in the workplace [22, 23]. Given the changes in workplace cultures, substance types, and usage patterns over time, more recent studies are needed to capture the evolving landscape of substance use in the workforce. Second, there is limited understanding of the specific characteristics of individuals engaging in substance use in the workplace besides basic demographic characteristics such as age and gender [24]. A more nuanced understanding—including work‐related and health‐related characteristics—would be essential for informing more effective and targeted interventions and workplace policies. Lastly, few studies examine how substance use risks vary across occupations. Without detailed investigations into the variations of substance use across occupations, significant gaps remain in our understanding of substance use in the workplace and its implications

To address these gaps, the present study utilizes data from a nationally representative longitudinal survey to examine the prevalence of substance use in the workplace, separately for alcohol, marijuana, and cocaine/hard drugs, and the characteristics of individuals engaging in these behaviors. Then, we evaluate the risks of substance use in the workplace across occupations and the specific needs of different worker groups.

## 2 Materials and Methods

### 2.1 Data and Sample

Data were drawn from the NLSY97, an ongoing prospective study that follows a nationally representative sample of birth cohorts born between 1980 and 1984. Since the baseline survey conducted in 1997 when participants were aged 12–16, The NLSY97 has collected data on labor market characteristics (e.g., employment status and work‐related factors), physical and behavioral health (e.g., substance use behaviors, up until the 2015–2016 survey), and individual and family characteristics. The NLSY97 employs a stratified multistage area probability sample design, with a baseline sample of 8,986 respondents recruited from 147 primary sampling units, each comprising a metropolitan area or one or more nonmetropolitan counties with at least 2000 housing units, selected from the National Opinion Research Center's 1990 master probability sample. Of the 7130 respondents (79.3% retention rate) who participated in the 2015–2016 survey—the most recent wave to collect data on substance use behaviors—our study focuses on 5827 participants who were employed and were not enrolled in school at the time of the survey. The final analytic sample was further restricted to 5465 after excluding 362 (6.2%) respondents with incomplete data. Additional details about NLSY97's study design and procedures are available from the BLS (https://www.bls.gov/nls/nlsy97.htm). The institution of the corresponding author, where the data analysis was conducted, does not require institutional review board approval for this study, as it utilizes deidentified data and is not considered human subjects research.

### 2.2 Measures

#### 2.2.1 Substance Use in the Workplace

The outcome variables are binary variables indicating whether a respondent used substances (asked separately for any substance [alcohol, marijuana, or cocaine/hard drugs], alcohol, marijuana, and cocaine/hard drugs) right before or during work in the past 30 days.

#### 2.2.2 Occupation

The independent variable is the respondent's occupation, classified according to the Census 2002 Standard Occupational Classification (SOC) codes. A total of 22 occupation categories were created by grouping occupations consistent with the 2002 SOC major occupational category structure.

#### 2.2.3 Covariates

We adjusted for sociodemographic characteristics (sex, race/ethnicity, highest degree received, marital status, annual household income), work‐related characteristics (underemployment status [defined as 15 or more weeks of unemployment in the past year, based on the U‐1 measure used by BLS], self‐employment status, work schedule types, union or employee association contract coverage status), and health‐related characteristics (general health condition, mental health condition [based on a five‐item short version of the Mental Health Inventory, developed by Veit and Ware [25]]).

### 2.3 Analytic Strategy

Statistical analyses were conducted in four steps. First, we estimated the prevalence of substance use in the workplace for the overall sample and by occupation, separately for any substance, alcohol, marijuana, and cocaine/hard drugs. Second, we examined the sociodemographic, work‐related, and health‐related characteristics of respondents who reported substance use in the workplace and those who did not. Third, we employed multivariable Poisson regression with robust standard errors to test the associations between occupation and substance use in the workplace, analyzed separately by substance type. Although logistic regression is commonly used for binary outcomes, we opted for Poisson regression with robust standard errors, as it tends to perform better, especially with cross‐sectional data when outcomes are not rare. Additionally, Poisson regression offers more intuitive interpretation through incidence rate ratios, compared to the odds ratios produced by logistic regression. In addition to our main models that included sociodemographic and health‐related covariates, we conducted supplemental analyses using a hierarchical approach to assess the robustness of the estimates and the impact of different sets of covariate sets. Specifically, we estimated our models: Model 1 included no covariates; Model 2 included only sociodemographic covariates; Model 3 included only health‐related covariates; Model 4 included all covariates, including work‐related characteristics. Finally, we estimated the predicted prevalence for each occupation based on the estimated coefficients from the main models. All estimates were weighted to account for the NLSY97' s stratified multistage area probability sample design.

## 3 Results

### 3.1 Prevalence and Characteristics of Respondents Reporting Past‐Month Substance Use in the Workplace

Table 1 presents the prevalence of substance use in the workplace among respondents and their characteristics. Overall, 8.9% (95% CI = 8.1–9.7) of respondents reported using any substance in the workplace. Additionally, 5.9% (95% CI = 5.3–6.6), 3.1% (95% CI = 2.7–3.7) and 0.8% (95% CI = 0.5–1.1) of respondents reported using alcohol, marijuana, and cocaine/hard drugs, respectively. Compared to their non‐using counterparts, respondents who reported any substance use in the workplace were more likely to be male, Black or Hispanic, have less than a high school education (vs. a Bachelor's degree or higher), be never married (vs. married), have lower annual household income ($66,043 vs. $80,258), and work regular evening shifts (vs. regular day shifts). They also reported more days of heavy drinking (defined as consuming five or more drinks in a day: 3.4 days vs. 1.2 days), more days of past‐month marijuana use (11.0 days vs. 1.5 days), and more instances of illicit drug use in the past year (9.8 vs. 0.9). Similar patterns were observed for alcohol use in the workplace, except marital status and past‐year illicit drug use behaviors. For respondents reporting marijuana use in the workplace, individuals were more likely to be male, be Black, have lower annual household income, and engage in more days of heavy drinking (3.4 days vs. 1.4 days) and notably more days of marijuana use (26.0 days vs. 1.5 days) in the past month compared to their non‐using counterparts. For respondents reporting cocaine/hard drug use in the workplace, individuals were more likely to have lower annual household income, be underemployed, and engage in more days of heavy drinking (6.2 days vs. 1.4 days) and marijuana use (8.2 days vs. 2.3 days) in the past months, and instances of illicit drug use in the past year (111.5 times vs. 0.9 times) compared to their non‐using counterparts.

**Table 1:** Sociodemographic, work‐related, and health‐related characteristics of respondents reporting past‐month substance use in the workplace among US Workers aged 30–35 in 2015–2016; National Longitudinal Survey of Youth 1997.

	Any (*n* = 540; 8.9%)		Alcohol (*n* = 383; 5.9%)		Marijuana (*n* = 171; 3.1%)		Cocaine/Hard Drugs (*n* = 36; 0.8%)	
	%, µ	95% CI	%, µ	95% CI	%, µ	95% CI	%, µ	95% CI
Sociodemographic characteristics								
Sex								
Male	10.5	9.4–11.8	6.8	5.9–7.8	4.0	3.3–4.8	1.1	0.7–1.6
Female	7.0	6.0–8.1	4.9	4.1–5.8	2.1	1.6–2.8	0.3	0.2–0.7
Race/ethnicity								
Black	14.2	12.3–16.4	10.5	8.9–12.4	4.8	3.6–6.3	0.5	0.2–1.6
Hispanic	11.0	9.2–13.0	8.1	6.6–9.9	2.9	2.1–4.2	1.0	0.5–1.9
Multi‐racial	13.4	6.5–25.6	9.2	3.9–20.3	4.2	1.1–15.3	0	—
White	7.4	6.5–8.4	4.6	3.9–5.4	2.8	2.3–3.5	0.8	0.5–1.2
Highest degree received								
None	12.7	9.4–16.9	7.6	5.2–11.1	5.3	3.2–8.5	1.3	0.5–3.7
High school	10.5	9.4–11.8	6.8	5.9–7.8	3.9	3.2–4.7	1.0	0.7–1.5
Associate degree	7.5	5.2–10.7	5.6	3.7–8.4	2.2	1.1–4.4	1.1	0.4–2.9
Bachelor's degree	6.8	5.4–8.5	4.4	3.3–5.8	2.5	1.7–3.6	0.1	0.0–0.7
Graduate degree	5.2	3.5–7.5	4.5	3.0–6.6	0.7	0.2–2.1	0.5	0.1–1.9
Current marital status								
Never married	11.5	10.1–12.9	6.9	5.9–8.0	5.0	4.0–6.1	1.1	0.7–1.7
Married	6.7	5.7–7.8	5.1	4.3–6.1	1.7	1.2–2.3	0.3	0.2–0.7
Separated/Divorced/Widowed	9.7	7.4–12.6	6.0	4.3–8.4	3.1	1.9–5.1	1.5	0.7–3.2
Number of children in household								
0	9.9	8.6–11.4	6.1	5.1–7.3	3.9	3.1–4.9	1.1	0.7–1.8
1	8.1	6.5–10.0	5.8	4.5–7.5	2.4	1.6–3.6	0.5	0.2–1.2
2+	8.4	7.2–9.6	5.8	4.9–6.8	2.7	2.1–3.6	0.5	0.3–1.0
Annual household Income (in $1,000)	66.0	59.9–72.2	66.9	59.8–73.9	60.0	49.5–70.6	62.8	39.6–86.0
Work‐related characteristics								
Underemployed (past‐year)								
No	8.6	7.8–9.5	5.7	5.1–6.5	3.1	2.6–3.7	0.6	0.4–0.9
Yes	10.6	8.6–12.9	6.9	5.3–8.8	3.4	2.3–4.9	1.5	1.0–2.4
Self‐employment status								
No	8.7	7.9–9.6	5.8	5.2–6.5	3.0	2.6–3.6	0.8	0.5–1.1
Yes	12.7	8.9–17.9	7.8	4.9–11.9	4.7	2.5–8.7	0.6	0.1–3.8
Work schedule type								
Regular: Day	8.1	7.1–9.1	5.7	4.9–6.5	2.7	2.2–3.4	0.4	0.2–0.7
Regular: Evening	15.2	11.1–20.5	11.2	7.7–16.1	5.2	3.0–8.8	1.5	0.5–4.5
Regular: Night	13.1	9.1–18.4	7.7	4.7–12.4	6.4	3.7–10.8	1.1	0.2–5.1
Rotation/Split	7.2	5.4–9.4	3.9	2.7–5.6	3.1	2.0–4.9	1.3	0.6–2.6
Irregular	10.0	6.5–14.9	6.0	3.5–10.2	3.6	1.7–7.2	1.2	0.3–4.5
Contract coverage by union or employee association								
No	8.5	7.6–9.6	5.7	5.0–6.5	3.0	2.4–3.6	0.7	0.5–1.1
Yes	7.4	5.6–9.9	5.7	4.0–7.9	2.1	1.2–3.7	0.3	0.1–2.2
Health‐related characteristics								
General Health Condition (range: 1–5)	3.6	3.5–3.7	3.7	3.6–3.8	3.4	3.2–3.6	3.7	3.4–4.0
Mental Health (range: 1–4)	2.0	2.0–2.1	2.0	2.0–2.1	2.1	2.0–2.1	2.0	2.0–2.1
# days of 5+ drinks per day in the past month	3.4	2.8–4.0	3.8	3.0–4.6	3.4	2.5–4.4	6.2	3.0–9.3
# days of marijuana use in the past month	11.0	9.7–12.2	4.8	3.7–5.9	26.0	24.8–27.2	8.2	4.1–12.3
# times of illicit drug use in the past year	9.8	4.8–14.8	2.6	0.1–5.7	7.5	0.1–15.2	111.5	62.9–160.0

*Note:* All percentages and mean estimates have been adjusted using sampling weights.

### 3.2 Prevalence of Past‐Month Substance Use in the Workplace by Occupation

Table 2 shows that individuals in food preparation/serving occupations reported the highest prevalences for all substances: 15.5% (95% CI = 11.7–20.3) for any substance, 8.8% (95% CI = 6.0–12.5) for alcohol, 8.1% (95% CI = 5.3–12.2) for marijuana, and 3.1% (95% CI = 1.6–6.0) for cocaine/hard drugs. For any substance use, the next highest prevalences were found in transportation/material moving (11.8%), installation/maintenance/repairs (11.7%), and arts/entertainment/recreation (11.0%). For alcohol use, the next highest prevalences were found in transportation/material moving (7.6%), scientists/related workers (7.5%), cleaning (7.5%), and setter/operators/tenders (7.4%). For marijuana use, the next highest prevalences were observed in installation/maintenance/repairs (4.5%), sales (4.4%), construction/extraction (4.3%), and arts/entertainment/recreation (4.1%) occupations. For cocaine/hard drug use, legal (2.1%), setter/operators/tenders (1.8%), and construction/extraction (1.4%) occupations had the highest prevalences after food preparation/serving occupations.

**Table 2:** Sample size and weighted prevalence of past‐month substance use in the workplace among US workers aged 30–35, by occupation in 2015–2016; National Longitudinal Survey of Youth 1997.

	Any			Alcohol			Marijuana			Cocaine/Hard Drugs		
	*n*	%	95% CI	*n*	%	95% CI	*n*	%	95% CI	*n*	%	95% CI
Executive/admin/managerial (*n* = 513)	52	9.4	7.1–12.4	36	6.3	4.5–8.9	19	3.6	2.2–5.7	1	0.2	0.1–1.6
Management (*n* = 249)	18	6.4	3.9–10.3	10	3.6	1.8–6.9	7	2.5	1.1–5.4	1	0.4	0.1–2.5
Scientists/related workers (*n* = 245)	28	9.8	6.6–14.3	23	7.5	4.8–11.6	5	2.2	0.9–5.3	1	0.6	0.1–4.0
Counselor/social/religious workers (*n* = 109)	8	7.0	3.2–14.6	7	6.1	2.6–13.7	1	0.9	0.1–5.9	0	0	—
Legal (*n* = 58)	2	3.4	0.8–13.2	1	1.3	0.2–8.8	0	0	—	1	2.1	0.3–13.5
Education/related workers (*n* = 297)	17	4.8	2.9–8.0	13	3.5	1.9–6.3	4	1.4	0.5–3.8	0	0	—
Arts/entertainment/recreation (*n* = 137)	19	11.0	6.7–17.5	12	5.9	3.2–10.7	6	4.1	1.7–9.7	1	1.0	0.1–6.6
Healthcare (*n* = 441)	29	5.0	3.3–7.5	22	3.5	2.1–5.7	7	1.3	0.6–2.9	1	0.3	0.0–2.2
Protective services (*n* = 151)	9	4.2	2.0–8.6	8	3.4	1.5–7.3	1	0.8	0.1–5.6	0	0	—
Food preparation/serving (*n* = 319)	55	15.5	11.7–20.3	34	8.8	6.0–12.5	25	8.1	5.3–12.2	9	3.1	1.6–6.0
Cleaning (*n* = 185)	23	10.4	6.6–16.2	19	7.5	4.5–12.3	6	3.4	1.4–8.2	1	0.9	0.1–5.8
Entertainment attendants/related workers (*n* = 25)	1	5.0	0.7–28.0	1	5.0	0.7–28.0	0	0	—	0	0	—
Personal care/services (*n* = 211)	17	6.5	3.8–11.1	13	4.6	2.4–8.6	5	2.3	0.8–6.1	1	0.8	0.1–5.1
Sales (*n* = 534)	59	10.5	8.0–13.7	39	6.3	4.5–8.9	22	4.4	2.8–6.8	4	0.8	0.3–2.2
Office/admin support services (*n* = 755)	69	8.9	6.9–11.5	53	6.5	4.8–8.7	18	2.6	1.6–4.3	3	0.4	0.1–1.4
Farming/fishing/forestry (*n* = 29)	1	1.2	0.2–7.9	1	1.2	0.2–7.9	0	0	0	0	0	0
Construction/extraction (*n* = 318)	35	10.0	7.1–14.1	21	5.7	3.6–8.9	14	4.3	2.5–7.4	5	1.4	0.5–3.6
Installation/maintenance/repairs (*n* = 183)	23	11.7	7.7–17.5	16	7.2	4.2–11.9	8	4.5	2.2–9.1	1	0.5	0.1–3.6
Production/operations (*n* = 77)	5	4.1	1.6–10.0	5	4.1	1.6–10.0	0	0	—	0	0	—
Setter/operators/tenders (*n* = 217)	19	8.2	5.0–13.3	15	7.4	4.3–12.5	6	1.9	0.7–5.3	3	1.8	0.5–6.0
Transportation/material moving (*n* = 399)	50	11.8	8.8–15.7	33	7.6	5.2–10.9	17	4.0	2.3–6.7	3	1.0	0.3–3.1
Armed Forces (*n* = 13)	1	3.6	0.5–22.6	1	3.6	0.5–22.6	0	0	—	0	0	—
All (*n* = 5465)	540	8.9	8.1–9.7	383	5.9	5.3–6.6	171	3.1	2.7–3.7	36	0.8	0.5–1.1

*Note:* The occupation categories are based on the US Census Bureau's 2002 four‐digit occupation classification (SOC). Sample sizes for occupations and cell frequencies are unweighted. All percentages and 95% confidence intervals have been weighted to account for the complex sampling design of the NLSY97.

### 3.3 Associations Between Occupations and Past‐Month Substance Use in the Workplace

Table 3 presents the estimates from the multivariable logistic regression of past‐month substance use in the workplace for individuals in different occupations compared to healthcare‐related occupations (the reference group) which had relatively lower prevalences and a large sample size to ensure more robust comparisons. For any substance use, individuals employed in food preparation/serving (IRR = 2.13, 95% CI = 1.29–3.53), arts/entertainment/recreation (IRR = 1.99, 95% CI = 1.08–3.68), scientists and related (IRR = 1.93, 95% CI = 109–3.43), and Executive/admin/managerial (IRR = 1.89, 95% CI = 1.13–3.14) occupations were more likely to report any substance use in the workplace in the past month than those in healthcare‐related occupations. For alcohol use, individuals with scientists and related (IRR = 2.26, 95% CI = 1.16–4.43), food preparation/serving (IRR = 2.00, 95% CI = 1.06–3.78), and executive/admin/managerial (IRR = 1.95, 95% CI = 1.04–3.66) occupations were more likely to consume alcohol in the workplace in the past month than those with healthcare‐related occupations. For marijuana use, individuals with food preparation/serving (IRR = 3.30, 95% CI = 1.17–9.27) and executive/administrative/managerial (IRR = 2.52, 95% CI = 1.01–6.30) were more likely to report past‐month marijuana use in the workplace than those with healthcare‐related occupations. For cocaine/hard drugs, although no occupations attained statistical significance, likely due to the rarity of the event, individuals with food preparation/serving (IRR = 4.88, 95% CI = 0.48–49.97) and legal (IRR = 4.60, 95% CI = 0.40–52.87) occupations indicated a positive association with cocaine/hard drug use in the workplace. Supplemental analyses using different sets of covariates (i.e., sociodemographic, health, and work‐related characteristics) showed minimal variation in the estimates once sociodemographic factors were accounted for (see Tables S1–S4).

**Table 3:** Associations Between Occupations and Past‐Month Substance Use in the Workplace Among US Workers Aged 30–35; National Longitudinal Survey of Youth 1997.

	Any		Alcohol		Marijuana		Cocaine/Hard Drugs	
	IRR	95% CI	IRR	95% CI	IRR	95% CI	IRR	95% CI
Healthcare‐related (Reference)	1.00	—	1.00	—	1.00	—	1.00	—
Executive/admin/managerial	**1.89**	**1.13–3.14**	**1.95**	**1.04–3.66**	**2.52**	**1.01–6.30**	0.50	0.03–8.79
Management	1.36	0.76–2.42	1.16	0.51–2.62	1.88	0.63–5.59	0.95	0.05–16.89
Scientists/related workers	**1.93**	**1.09–3.43**	**2.26**	**1.16–4.43**	1.49	0.50–4.48	1.23	0.09–17.81
Counselor/social/religious workers	1.48	0.60–3.67	1.78	0.64–4.98	0.79	0.09–7.05	—	—
Legal	0.73	0.16–3.27	0.37	0.04–3.10	—	—	4.60	0.40–52.87
Education/related workers	1.12	0.56–2.22	1.10	0.51–2.37	1.39	0.37–5.25	—	—
Arts/entertainment/recreation	**1.99**	**1.08–3.68**	1.66	0.67–4.13	2.58	0.90–7.40	1.82	0.11–30.96
Protective services	0.62	0.28–1.35	0.77	0.33–1.84	0.41	0.05–3.15	—	—
Food preparation/serving	**2.13**	**1.29–3.53**	**2.00**	**1.06–3.78**	**3.30**	**1.17–9.27**	4.88	0.48–49.97
Cleaning	1.32	0.71–2.47	1.54	0.71–3.35	1.35	0.49–3.74	1.13	0.06–20.16
Entertainment attendants/related workers	0.78	0.11–5.81	1.31	0.18–9.74	—	—	—	—
Personal care/services	1.09	0.50–2.36	1.16	0.50–2.68	1.37	0.34–5.54	2.16	0.12–38.84
Sales	**1.72**	**1.01–2.91**	1.66	0.90–3.08	2.28	0.93–5.60	1.47	0.14–15.70
Office/admin support services	1.51	0.95–2.42	1.69	0.93–3.10	1.53	0.61–3.85	0.86	0.07–10.03
Farming/fishing/forestry	0.18	0.02–1.44	0.32	0.04–2.62	—	—	—	—
Construction/extraction	1.34	0.77–2.31	1.27	0.65–2.48	1.70	0.65–4.46	1.42	0.12–16.30
Installation/maintenance/repairs	1.68	0.94–2.99	1.71	0.78–3.76	1.91	0.65–5.62	0.54	0.03–11.11
Production/operations	0.55	0.19–1.57	0.89	0.30–2.64	—	—	—	—
Setter/operators/tenders	1.09	0.56–2.12	1.63	0.77–3.46	0.74	0.20–2.74	2.20	0.18–27.14
Transportation/material moving	1.51	0.91–2.51	1.60	0.86–2.96	1.50	0.62–3.65	1.25	0.11–14.85
Armed Forces	0.61	0.08–4.61	0.92	0.12–6.86	—	—	—	—

*Note:* The occupation categories are based on the US Census Bureau's 2002 Standard Occupational Classification (SOC) codes. Covariates included sex, race/ethnicity, highest degree received, marital status, annual household income, general health, and mental health condition. Incidence Rate Ratios (IRRs) and 95% CI for the covariates are not presented to conserve space. Bolded IRRs indicate statistical significance at the 0.05 level. No estimates are provided for occupations in which no respondents reported any use. The estimates and confidence intervals are adjusted for the complex sampling design using sample weight.

**Figure 1:**
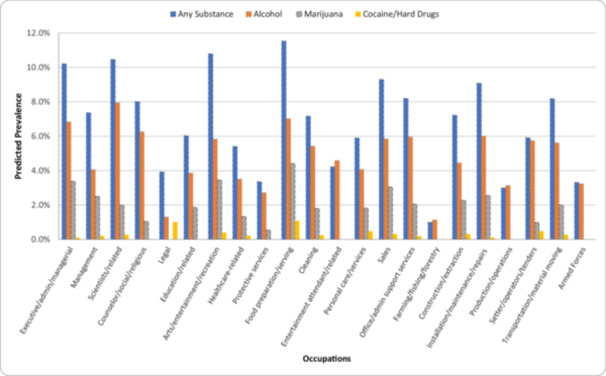
Predicted Prevalence of Past‐Month Substance Use in the Workplace among US Workforce Aged 30–35 by Occupation, National Longitudinal Survey of Youth 1997. *Note:* The predicted prevalence figures were estimated using the coefficients for each occupation obtained from the coefficient estimates from the main models in Table 3.

Figure 1 shows the estimated prevalence of past‐month workplace substance use among individuals with average values on the covariates included in the main models in Table 3. The estimates suggest that 11.5% of food preparation/serving workers, 10.8% of arts/entertainment/recreation workers, 10.5% of scientists and related workers, and 10.2% of executive/administrative/managerial workers are expected to use any substance in the workplace in the past month. For alcohol use, 7.9% of scientists/related workers, 7.0% of food preparation/serving workers, and 6.8% of executive/administrative/managerial workers are expected to use alcohol in the workplace in the past month. For marijuana use, 4.4% of food preparation/serving workers, 3.5% of arts/entertainment/recreation workers, 3.4% of executive/administrative/managerial workers, and 3.1% of sales workers are expected to engage in marijuana use. For cocaine/hard drugs, 1.1% of food preparation/serving workers and 1.0% of legal workers are expected to engage in use.

## 4 Discussion

This study used a nationally representative sample of workers in their early 30 s, a group at heightened risk of substance use and active workforce participation, to examine the prevalence of substance use behaviors that can directly and indirectly impact health, safety, and performance in the workplace. Our findings suggest that nearly nine out of every ten workers in their early 30s reported using alcohol, marijuana, or cocaine/hard drugs in the workplace within the past month, while 5.9% used alcohol, 3.1% used marijuana, and 0.8% used cocaine/hard drugs. These behaviors raise significant concerns about the health and safety of users, as well as the economic and social costs associated with them. Overall, we found that workers who were nonwhite or from lower socioeconomic backgrounds (e.g., without a high school education or with low household income) faced greater risks of substance use in the workplace, with some variations across substance types. Additionally, individuals who used marijuana in the workplace were more than twice as likely to engage in heavy drinking compared to those who did not, and they used marijuana nearly every day. Among those who used cocaine/hard drugs in the workplace, general substance use was more severe: they engaged in heavy drinking more than four times as often, used marijuana more than three times as frequently, and reported over 111 instances of illicit drug use in the past year. This highlights significant health risks among those using marijuana or cocaine/hard drugs in the workplace and the associated costs for workplaces, healthcare systems, and society. These findings underscore the importance of providing access to cost‐effective workplace‐based services for individuals engaging in substance use in the workplace, given the extent and persistence of their use in and outside of the workplace [26].

Substance use in the workplace varied widely across occupations, consistent with general patterns of substance use across occupations [19]. Specifically, the prevalence of substance use in the workplace was highest in food preparation/serving occupations for all substance types examined. The incident rate for individuals with food preparation and serving occupations—while controlling for sociodemographic and health‐related characteristics—was more than two times the incident rate for the reference group (i.e., healthcare‐related workers) for any substance use and more than three times the incident rate for marijuana use in the workplace compared to those with healthcare‐related occupations. Food preparation/serving workers, often exposed to adverse working conditions and long hours, face higher risks of substance use for coping with physical and emotional stress, easier access to substances in the workplace, and permissive norms [27, 28].

We also found that white‐collar workers [29], especially those with scientists/related and executive/administrative/managerial occupations, reported greater use of alcohol in the workplace. Prior research suggests that these occupations, especially management, often involve alcohol‐involved work‐related activities (such as lunch) and leisure activities, given their higher disposable income [14, 15, 16, 17]. Among managers, for example, alcohol consumption is often viewed as an essential part of building business relationships, which can increase both the opportunities to drink and the pressure to do so with colleagues and partners [14]. Also, scientists have reported the pervasiveness of alcohol—not only at university happy hours, conferences, and during fieldwork, but also in labs and other scientific settings to celebrate achievements and facilitate networking, although this trend is perceived to be declining in recent years [30].

However, it was particularly alarming to observe higher levels of substance use in the workplace among workers with safety‐sensitive occupations—especially skilled trades and transportation/material moving [19]. Specifically, our models estimated that around 8% of workers in installation/maintenance/repair, transportation/material moving, and construction/extraction occupations were expected to use any substances in the workplace, with 6% using alcohol and over 2% using marijuana. These occupations often involve high stress due to chronic physical demands, exposure to high occupational risks, and hazardous working conditions while having workplace cultures that normalize substance use [31, 32]. Although an increasing number of workplaces have established policies prohibiting substance use at work and off‐duty use of controlled substances since the introduction of the Drug‐Free Workplace Act of 1988 (see Frone and Bamberger [33]), evidence suggests the importance of a multi‐pronged strategy is essential, including the provision of prevention and rehabilitative services [33, 34, 35]. While evidence on substance use in the workplace—particularly among workers in safety‐sensitive occupations—is limited, research suggests that individuals employed in settings with comprehensive approaches, including the implementation of alcohol and drug policies, testing, and supportive services (e.g., access to employee assistance programs or counseling for substance‐related problems), are significantly less likely to engage in substance use compared to those in workplaces that offer only one of these components or none at all [34, 35]. However, the transient nature of these jobs, i.e., working in the field or at temporary sites, makes implementing an effective program challenging and thereby highlights the need for interventions tailored to specific occupational settings to effectively reduce substance use [18, 22].

There are several limitations to note when interpreting these findings. First, our study does not warrant a causal relationship, particularly between occupation type and substance use behaviors. Longitudinal data and quasi‐experimental designs would be needed to address potential biases, such as omitted variable bias or reverse causality. Second, this study does not explore the mechanisms behind why certain occupations are at greater risk for substance use in the workplace. Information on workplace contexts—such as substance use norms, physical or emotional stress, and substance‐related policies—would provide valuable insights into these mechanisms. Third, the rarity of some of the events studied, particularly past‐month cocaine/hard drug use in the workplace, led to larger standard errors and a lack of statistical significance. However, non‐significance does not necessarily imply that there are no differences in cocaine/hard drug use across occupations, indicating the need for future research with larger sample sizes (with or without oversampled individuals from high‐risk occupations) or a more focused examination of specific occupations. Fourth, our results may be subject to underreporting due to social desirability bias, which could also vary across occupations. Although NLSY97 respondents entered their answers directly into a laptop during sensitive portions of the interview, potentially reducing social desirability bias, differences in personality or situational factors across workers in different occupations may still influence the reporting of sensitive information. Lastly, our measures do not distinguish prescription or synthetic opioid use from the cocaine/hard drugs category. Future research is needed to better understand opioid use in the workplace, as it may help explain the sharp rise in fatal workplace injuries since the early 2010 s.

Despite the limitations, this study provides valuable evidence to inform research and intervention strategies for a significant public health issue using recent data from a nationally representative sample of young workers in the United States. The high prevalence of substance use in the workplace raises, especially considering their notably frequent use of substances, concerns for the safety of the users, their colleagues, andothers exposed to the workplace or activities. Particularly concerning is the higher risk of alcohol and marijuana use in occupations with safety implications, which underscores the need for multi‐pronged intervention approaches to prevent and assist with rehabilitation for individuals engaged in persistent substance use both in and outside of the workplace.

## Author Contributions

**Sehun Oh:** conceptualization, data curation, funding acquisition, investigation, methodology, formal analysis, writing – original draft. **Daejun Park:** conceptualization, writing – review and editing. **Sarah Al‐Hashemi:** writing – original draft, writing – review and editing.

## Disclosure by AJIM Editor of Record

Jian Li declares that he has no conflicts of interest in the review and publication decision regarding this article.

## Ethics Statement

The Ohio State University, where the data analysis was conducted, does not require institutional review board approval for this study using publically‐available data, which is not considered human subjects research.

## Conflicts of Interest

The authors declare no conflicts of interest.

## Supporting information

SupportingDocument.

## Data Availability

The data that support the findings of this study are openly available in the National Longitudinal Surveys at https://www.nlsinfo.org/content/getting-started/accessing-data.
