# Supplementary material for: Substance Use Right Before or During Work Among the Young US Workers: Evidence From the National Longitudinal Survey of Youth 1997 Cohort
Source: Am J Ind Med. 2025 May 15;68(8):679–87. doi: 10.1002/ajim.23737 (PMC12242107; doi:10.1002/ajim.23737)
Supplement: Supplementary file 1 — SupportingDocument. [file AJIM-68-679-s001.docx]

Table S1. Associations between Occupations and Past-Month Any Substance Use in the Workplace among U.S. Workers of Ages 30-35, National Longitudinal Survey of Youth 1997

|  | M1: No Covariates | | M2: Sociodemographics | | M3: Health | | M3: All | |
| --- | --- | --- | --- | --- | --- | --- | --- | --- |
|  | IRR | 95% CI | IRR | 95% CI | IRR | 95% CI | IRR | 95% CI |
| Healthcare-related (Reference) | 1.00 | - | 1.00 | - | 1.00 | - | 1.00 | - |
| Executive/admin/managerial | **1.88** | **1.15-3.07** | **1.86** | **1.12-3.08** | **1.95** | **1.20-3.19** | **1.94** | **1.17-3.20** |
| Management | 1.29 | 0.73-2.27 | 1.33 | 0.75-2.36 | 1.34 | 0.76-2.37 | 1.36 | 0.76-2.41 |
| Scientists/related workers | **1.96** | **1.08-3.53** | **1.92** | **1.09-3.39** | **2.06** | **1.14-3.70** | **1.96** | **1.10-3.48** |
| Counselor/social/religious workers | 1.39 | 0.56-3.47 | 1.47 | 0.60-3.60 | 1.45 | 0.57-3.67 | 1.51 | 0.61-3.73 |
| Legal | 0.69 | 0.14-3.25 | 0.76 | 0.17-3.43 | 0.68 | 0.15-3.21 | 0.73 | 0.16-3.27 |
| Education/related workers | 0.97 | 0.49-1.90 | 1.12 | 0.57-2.21 | 0.99 | 0.50-1.97 | 1.12 | 0.57-2.22 |
| Arts/entertainment/recreation | **2.20** | **1.18-4.08** | **1.98** | **1.07-3.67** | **2.23** | **1.21-4.13** | **1.88** | **1.03-3.43** |
| Protective services | 0.84 | 0.37-1.91 | 0.61 | 0.28-1.37 | 0.85 | 0.38-1.89 | 0.61 | 0.27-1.35 |
| Food preparation/serving | **3.09** | **1.90-5.05** | **2.14** | **1.30-3.53** | **2.94** | **1.79-4.83** | **2.12** | **1.28-3.50** |
| Cleaning | **2.08** | **1.13-3.84** | 1.28 | 0.69-2.36 | **2.06** | **1.10-3.83** | 1.25 | 0.67-2.32 |
| Entertainment attendants/related workers | 0.99 | 0.14-7.2 | 0.82 | 0.11-6.04 | 0.93 | 0.13-6.93 | 0.84 | 0.11-6.29 |
| Personal care/services | 1.31 | 0.61-2.81 | 1.08 | 0.49-2.34 | 1.30 | 0.61-2.80 | 1.05 | 0.48-2.27 |
| Sales | **2.10** | **1.25-3.52** | **1.71** | **1.01-2.87** | **2.10** | **1.24-3.55** | **1.74** | **1.03-2.94** |
| Office/admin support services | **1.78** | **1.14-2.79** | 1.53 | 0.96-2.42 | **1.72** | **1.09-2.73** | 1.49 | 0.93-2.38 |
| Farming/fishing/forestry | 0.23 | 0.03-1.77 | 0.18 | 0.02-1.42 | 0.23 | 0.03-1.80 | 0.19 | 0.02-1.45 |
| Construction/extraction | **2.00** | **1.15-3.49** | 1.29 | 0.75-2.23 | **2.05** | **1.17-3.58** | 1.32 | 0.76-2.28 |
| Installation/maintenance/repairs | **2.34** | **1.32-4.15** | 1.67 | 0.93-2.97 | **2.33** | **1.31-4.14** | 1.68 | 0.94-2.99 |
| Production/operations | 0.81 | 0.28-2.36 | 0.54 | 0.19-1.52 | 0.81 | 0.27-2.37 | 0.53 | 0.19-1.50 |
| Setter/operators/tenders | 1.64 | 0.87-3.11 | 1.11 | 0.58-2.14 | 1.57 | 0.82-2.99 | 1.05 | 0.54-2.04 |
| Transportation/material moving | **2.35** | **1.45-3.81** | 1.51 | 0.92-2.49 | **2.28** | **1.39-3.74** | 1.49 | 0.90-2.47 |
| Armed Forces | 0.72 | 0.94-5.48 | 0.57 | 0.08-4.24 | 0.80 | 0.11-6.13 | 0.55 | 0.07-4.13 |

*Notes*. The occupation categories are based on the U.S. Census Bureau's 2002 Standard Occupational Classification (SOC) codes. All covariates included sociodemographic, health, and work-related characteristics. Incidence-Rate Ratios (IRRs) and 95% CI for the covariates are not presented to conserve space. Bolded IRRs indicate statistical significance at the 0.05 level. The estimates and confidence intervals are adjusted for the complex sampling design using sample weight.

Table S2. Associations between Occupations and Past-Month Alcohol Use in the Workplace among U.S. Workers of Ages 30-35, National Longitudinal Survey of Youth 1997

|  | M1: No Covariates | | M2: Sociodemographics | | M3: Health | | M3: All | |
| --- | --- | --- | --- | --- | --- | --- | --- | --- |
|  | IRR | 95% CI | IRR | 95% CI | IRR | 95% CI | IRR | 95% CI |
| Healthcare-related (Reference) | 1.00 | - | 1.00 | - | 1.00 | - | 1.00 | - |
| Executive/admin/managerial | 1.81 | 0.99-3.31 | **1.93** | **1.03-3.63** | **1.85** | **1.01-3.39** | **2.00** | **1.07-3.76** |
| Management | 1.03 | 0.46-2.32 | 1.14 | 0.50-2.57 | 1.06 | 0.47-2.39 | 1.12 | 0.49-2.55 |
| Scientists/related workers | **2.15** | **1.10-4.23** | **2.26** | **1.16-4.41** | **2.21** | **1.12-4.35** | **2.26** | **1.13-4.49** |
| Counselor/social/religious workers | 1.74 | 0.62-4.93 | 1.76 | 0.64-4.90 | 1.79 | 0.63-5.13 | 1.80 | 0.65-5.00 |
| Legal | 0.38 | 0.04-3.19 | 0.38 | 0.05-3.14 | 0.37 | 0.04-3.16 | 0.37 | 0.04-3.05 |
| Education/related workers | 1.00 | 0.45-2.19 | 1.10 | 0.51-2.37 | 1.01 | 0.46-2.22 | 1.06 | 0.49-2.30 |
| Arts/entertainment/recreation | 1.69 | 0.68-4.22 | 1.67 | 0.67-4.16 | 1.69 | 0.68-4.19 | 1.61 | 0.67-3.86 |
| Protective services | 0.96 | 0.40-2.30 | 0.77 | 0.32-1.85 | 0.97 | 0.41-2.27 | 0.75 | 0.32-1.78 |
| Food preparation/serving | **2.50** | **1.33-4.71** | **2.01** | **1.06-3.80** | **2.42** | **1.28-4.58** | **2.02** | **1.08-3.77** |
| Cleaning | **2.15** | **1.02-4.56** | 1.51 | 0.70-3.27 | **2.14** | **1.00-4.59** | 1.44 | 0.66-3.12 |
| Entertainment attendants/related workers | 1.42 | 0.19-10.5 | 1.39 | 0.19-10.3 | 1.34 | 0.18-10.2 | 1.44 | 0.19-10.8 |
| Personal care/services | 1.31 | 0.56-3.07 | 1.16 | 0.50-2.68 | 1.31 | 0.56-3.06 | 1.13 | 0.49-2.59 |
| Sales | 1.81 | 0.98-3.34 | 1.66 | 0.90-3.07 | 1.81 | 0.97-3.35 | 1.70 | 0.92-3.16 |
| Office/admin support services | **1.86** | **1.03-3.37** | 1.70 | 0.93-3.10 | 1.82 | 1.00-3.31 | 1.64 | 0.89-3.01 |
| Farming/fishing/forestry | 0.33 | 0.04-2.61 | 0.32 | 0.04-2.62 | 0.33 | 0.04-2.62 | 0.33 | 0.04-2.65 |
| Construction/extraction | 1.62 | 0.82-3.18 | 1.25 | 0.64-2.44 | 1.64 | 0.83-3.23 | 1.24 | 0.62-2.46 |
| Installation/maintenance/repairs | 2.05 | 0.93-4.50 | 1.71 | 0.78-3.77 | 2.04 | 0.93-4.46 | 1.68 | 0.76-3.72 |
| Production/operations | 1.16 | 0.38-3.53 | 0.88 | 0.30-2.59 | 1.16 | 0.38-3.56 | 0.84 | 0.28-2.45 |
| Setter/operators/tenders | 2.11 | 1.02-4.39 | 1.65 | 0.78-3.48 | 2.06 | 0.99-4.28 | 1.54 | 0.73-3.25 |
| Transportation/material moving | **2.17** | **1.20-3.93** | 1.60 | 0.87-2.94 | **2.13** | **1.16-3.91** | 1.57 | 0.85-2.91 |
| Armed Forces | 1.03 | 0.13-8.01 | 0.89 | 0.12-6.54 | 1.11 | 0.14-8.63 | 0.82 | 0.11-6.15 |

*Notes*. The occupation categories are based on the U.S. Census Bureau's 2002 Standard Occupational Classification (SOC) codes. All covariates included sociodemographic, health, and work-related characteristics. Incidence-Rate Ratios (IRRs) and 95% CI for the covariates are not presented to conserve space. Bolded IRRs indicate statistical significance at the 0.05 level. The estimates and confidence intervals are adjusted for the complex sampling design using sample weight.

Table S3. Associations between Occupations and Past-Month Marijuana Use in the Workplace among U.S. Workers of Ages 30-35, National Longitudinal Survey of Youth 1997

|  | M1: No Covariates | | M2: Sociodemographics | | M3: Health | | M3: All | |
| --- | --- | --- | --- | --- | --- | --- | --- | --- |
|  | IRR | 95% CI | IRR | 95% CI | IRR | 95% CI | IRR | 95% CI |
| Healthcare-related (Reference) | 1.00 | - | 1.00 | - | 1.00 | - | 1.00 | - |
| Executive/admin/managerial | **2.75** | **1.08-6.99** | 2.39 | 0.95-6.02 | **3.04** | **1.21-7.62** | **2.55** | **1.05-6.19** |
| Management | 1.92 | 0.67-5.53 | 1.80 | 0.61-5.30 | 2.11 | 0.74-5.99 | 1.99 | 0.67-5.86 |
| Scientists/related workers | 1.72 | 0.56-5.24 | 1.44 | 0.49-4.20 | 1.94 | 0.63-5.97 | 1.52 | 0.50-4.66 |
| Counselor/social/religious workers | 0.67 | 0.08-5.62 | 0.77 | 0.09-6.87 | 0.73 | 0.09-6.12 | 0.81 | 0.09-7.25 |
| Legal | - | - | - | - | - | - | - | - |
| Education/related workers | 1.05 | 0.28-3.97 | 1.35 | 0.36-5.09 | 1.14 | 0.30-4.33 | 1.46 | 0.40-5.36 |
| Arts/entertainment/recreation | **3.18** | **1.10-9.20** | 2.44 | 0.85-7.00 | **3.39** | **1.18-9.75** | 2.21 | 0.78-6.31 |
| Protective services | 0.64 | 0.07-5.45 | 0.39 | 0.05-3.04 | 0.67 | 0.08-5.73 | 0.41 | 0.05-3.34 |
| Food preparation/serving | **6.27** | **2.37-16.6** | **3.19** | **1.14-8.98** | **5.80** | **2.18-15.5** | **3.45** | **1.26-9.47** |
| Cleaning | 2.66 | 0.95-7.47 | 1.23 | 0.43-3.48 | 2.62 | 0.95-7.19 | 1.25 | 0.46-3.40 |
| Entertainment attendants/related workers | - | - | - | - | - | - | - | - |
| Personal care/services | 1.77 | 0.44-7.14 | 1.27 | 0.31-5.26 | 1.78 | 0.45-7.09 | 1.25 | 0.32-4.88 |
| Sales | **3.37** | **1.39-8.20** | 2.18 | 0.89-5.34 | **3.42** | **1.40-8.35** | 2.34 | 0.97-5.64 |
| Office/admin support services | 2.02 | 0.82-5.00 | 1.53 | 0.60-3.88 | 1.93 | 0.78-4.77 | 1.57 | 0.63-3.91 |
| Farming/fishing/forestry | - | - | - | - | - | - | - | - |
| Construction/extraction | **3.33** | **1.26-8.83** | 1.53 | 0.58-4.02 | **3.53** | **1.34-9.29** | 1.68 | 0.65-4.32 |
| Installation/maintenance/repairs | **3.50** | **1.21-10.2** | 1.83 | 0.62-5.40 | **3.52** | **1.21-10.2** | 1.92 | 0.65-5.63 |
| Production/operations | - | - | - | - | - | - | - | - |
| Setter/operators/tenders | 1.50 | 0.41-5.43 | 0.75 | 0.21-2.76 | 1.39 | 0.38-5.08 | 0.75 | 0.21-2.75 |
| Transportation/material moving | **3.07** | **1.33-7.11** | 1.47 | 0.60-3.57 | **2.92** | **1.27-6.72** | 1.52 | 0.63-3.66 |
| Armed Forces | - | - | - | - | - | - | - | - |

*Notes*. The occupation categories are based on the U.S. Census Bureau's 2002 Standard Occupational Classification (SOC) codes. All covariates included sociodemographic, health, and work-related characteristics. Incidence-Rate Ratios (IRRs) and 95% CI for the covariates are not presented to conserve space. Bolded IRRs indicate statistical significance at the 0.05 level. The estimates and confidence intervals are adjusted for the complex sampling design using sample weight.

Table S4. Associations between Occupations and Past-Month Cocaine/Hard Drug Use in the Workplace among U.S. Workers of Ages 30-35, National Longitudinal Survey of Youth 1997

|  | M1: No Covariates | | M2: Sociodemographics | | M3: Health | | M3: All | |
| --- | --- | --- | --- | --- | --- | --- | --- | --- |
|  | IRR | 95% CI | IRR | 95% CI | IRR | 95% CI | IRR | 95% CI |
| Healthcare-related (Reference) | 1.00 | - | 1.00 | - | 1.00 | - | 1.00 | - |
| Executive/admin/managerial | 0.71 | 0.04-11.63 | 0.50 | 0.03-8.73 | 0.71 | 0.04-11.81 | 0.67 | 0.04-12.19 |
| Management | 1.13 | 0.07-19.29 | 0.92 | 0.05-15.81 | 1.14 | 0.07-19.92 | 1.26 | 0.07-22.79 |
| Scientists/related workers | 1.83 | 0.12-29.02 | 1.23 | 0.08-18.19 | 1.84 | 0.12-28.86 | 1.65 | 0.09-29.19 |
| Counselor/social/religious workers | - | - | - | - | - | - | - | - |
| Legal | 6.68 | 0.41-110.04 | 4.69 | 0.40-55.49 | 6.57 | 0.41-106.43 | 4.69 | 0.51-43.46 |
| Education/related workers | - | - | - | - | - | - | - | - |
| Arts/entertainment/recreation | 3.07 | 0.18-51.09 | 1.85 | 0.11-31.06 | 3.04 | 0.18-50.93 | 2.05 | 0.12-34.62 |
| Protective services | **-** | **-** | - | - | - | - | - | - |
| Food preparation/serving | **9.79** | **1.17-81.88** | 4.90 | 0.47-50.56 | **9.65** | **1.16-80.19** | 3.87 | 0.37-40.57 |
| Cleaning | 2.69 | 0.16-43.74 | 1.12 | 0.06-19.93 | 2.69 | 0.16-44.25 | 1.33 | 0.08-22.88 |
| Entertainment attendants/related workers | - | - | - | - | - | - | - | - |
| Personal care/services | 2.35 | 0.14-39.25 | 2.16 | 0.12-37.88 | 2.35 | 0.14-39.25 | 2.46 | 0.13-46.89 |
| Sales | 2.51 | 0.26-24.22 | 1.47 | 0.14-15.83 | 2.51 | 0.26-24.33 | 1.49 | 0.13-17.61 |
| Office/admin support services | 1.21 | 0.11-13.07 | 0.86 | 0.07-10.12 | 1.20 | 0.11-12.99 | 1.01 | 0.08-12.76 |
| Farming/fishing/forestry | - | - | - | - | - | - | - | - |
| Construction/extraction | 4.45 | 0.48-41.38 | 1.42 | 0.12-16.62 | 4.47 | 0.48-41.62 | 1.99 | 0.17-23.94 |
| Installation/maintenance/repairs | 1.63 | 0.10-27.09 | 0.54 | 0.03-11.42 | 1.63 | 0.10-26.89 | 0.67 | 0.03-13.11 |
| Production/operations | - | - | - | - | - | - | - | - |
| Setter/operators/tenders | 5.60 | 0.54-58.38 | 2.25 | 0.18-27.84 | 5.55 | 0.53-58.48 | 2.26 | 0.17-29.94 |
| Transportation/material moving | 3.23 | 0.33-31.73 | 1.25 | 0.10-14.97 | 3.21 | 0.32-31.98 | 1.36 | 0.11-16.77 |
| Armed Forces | - | - | - | - | - | - | - | - |

*Notes*. The occupation categories are based on the U.S. Census Bureau's 2002 Standard Occupational Classification (SOC) codes. All covariates included sociodemographic, health, and work-related characteristics. Incidence-Rate Ratios (IRRs) and 95% CI for the covariates are not presented to conserve space. Bolded IRRs indicate statistical significance at the 0.05 level. The estimates and confidence intervals are adjusted for the complex sampling design using sample weight.
